# Journal of Medical Case Reports: the first 100 cases

**DOI:** 10.1186/1752-1947-2-168

**Published:** 2008-05-20

**Authors:** Michael R Kidd, Sara del Olmo Fernández, Deborah C Saltman

**Affiliations:** 1 Discipline of General Practice, The University of Sydney, Australia; 2 Trainee in Family and Communitarian Medicine, Madrid, Spain; 3 Institute of Postgraduate Medicine, Brighton and Sussex Medical School, UK

## 

The *Journal of Medical Case Reports *was launched in February 2007. This is an open access, online journal dedicated to the publication of high quality case reports which make a contribution to the expansion of medical knowledge [1]. It is the world's first international medical journal devoted to the publication of case reports from all areas of clinical medicine from anywhere in the world and accessible to all. The editors believe that properly described case reports can make a valuable contribution to medical research and education.

All articles published in the journal are freely and immediately available online. In addition all articles are archived in PubMed and PubMed Central. In line with the policy of the publisher, BioMed Central, an article processing charge is levied on all articles that are accepted for publication.

In the calendar year 2007, 185 peer-reviewed case reports were published in the journal. The content of the first 100 manuscripts accepted for publication has been analysed by clinical discipline, age and gender of the patients described in the case reports, country of origin of the patient and the first author of each manuscript, whether the authors claim that this was the first report of such a case published in the medical literature, whether the manuscript included clinical images, as well as further categorization of features of the case reports.

The most common clinical disciplines represented in the first 100 case reports were general surgery and general medicine each with 11 cases, followed by oncology (7), orthopaedics (7), ophthalmology (6), infectious diseases (6), emergency room (6), urology (5), paediatrics (5), haematology (4), otolaryngology (4), gastroenterology (3), intensive care (3), respiratory medicine (3), rheumatology (3), cardiology (2), clinical genetics (2), dermatology (2), endocrinology (2), gynaecology (2), vascular surgery (2), cardiothoracic surgery (1), nephrology (1), neurology (1) and radiology (1). There were no cases published from authors working in general practice among the first 100 case reports.

The age range of patients described in the case reports was 0–9 years (8 cases), 10–19 years (8), 20–39 years (24), 40–59 years (27), 60–75 years (23) and 75 years and above (10). There were 43 female patients and 57 male patients.

The majority of patients described in the case reports were living in Europe (49 cases) followed by Asia (24), North America (16), Africa (4), South America (4) and Australasia (3). The majority of patients were from the United Kingdom (30 cases) followed by the United States of America (14), Germany (5), Japan (5), Iran (4), Italy (4) and Pakistan (4).

The continent where the first author was based was Europe (51 cases), Asia (21), North America (21), Australasia (3), South America (3) and Africa (1). The majority of first authors were working in the United Kingdom (30), followed by the United States of America (17), Germany (5), Japan (5), Iran (4), Italy (4) and Pakistan (4).

31 cases were claimed by the author(s) to be the first ever report in the accessible medical literature of such a case. This demonstrates the contribution that case reports can make to the expansion of medical knowledge.

81 cases included clinical images. This demonstrates one of the great potential uses of the *Journal of Medical Case Reports *for medical education.

34 cases involved a person with an infection, 23 involved a person with cancer, 21 involved a person with a vascular condition, 14 involved a person with one or more malformations, 14 involved a person with a metabolic condition and 9 involved a person who had experienced trauma. 31 cases involved iatrogenic problems and 6 cases involved the death of the patient.

43 cases described a relatively common presentation of a rare condition, 27 cases described a rare presentation of a rare condition, 18 cases described a rare presentation of a common condition, and 12 cases described a relatively common presentation of a common condition but with specific features which made the case suitable for publication.

16 cases described unreported or unusual side effects or adverse interactions involving medications. 16 cases described unexpected or unusual presentations of a disease, 6 cases described new associations or variations in disease processes, 16 cases described presentation, diagnosis and/or management of new and emerging diseases, 20 cases described an unexpected association between diseases or symptoms, 24 cases described an unexpected event in the course of observing or treating a patient, and 2 cases described findings that shed new light on the possible pathogenesis of a disease or an adverse effect.

This analysis shows the great diversity in the cases being published in this journal with cases contributed from most areas of clinical medicine, from many different parts of the world and describing patients from infancy to old age.
